# *Waddlia chondrophila*, a Potential Agent of Human Fetal Death

**DOI:** 10.3201/eid1308.070315

**Published:** 2007-08

**Authors:** David Baud, Vincent Thomas, Aliaa Arafa, Lesley Regan, Gilbert Greub

**Affiliations:** * University of Lausanne, Lausanne, Switzerland; † St Mary’s Hospital, London, UK

**Keywords:** Miscarriage, abortion, Waddlia chondrophila, Chlamydia trachomatis, intracellular bacteria, chorioamnionitis, zoonosis, dispatch

## Abstract

We investigated the zoonotic potential of *Waddlia chondrophila*, a new *Chlamydia*-like abortigenic agent in ruminants. Anti-*Waddlia* antibody reactivity was tested by immunofluorescence and Western blot. *Waddlia* seroprevalence was higher in women who had had sporadic and recurrent miscarriages than in control women (p<0.001). *Waddlia* spp. may represent a cause of human fetal loss.

Approximately 15% of pregnancies end in miscarriage (*1*). However, a cause is identified in only 50% of these cases. Obligate intracellular bacteria, which fail to grow on media used routinely to isolate human pathogens, could represent yet unrecognized agents of miscarriage.

*Chlamydia trachomatis*, an intracellular bacterium, is the world’s most common sexually transmitted bacterial pathogen (*2*). Because *C. trachomatis* is asymptomatic, most infected women remain untreated. Although the effect of *C. trachomatis* infection on pregnancy outcome is unclear, an increased prevalence of *C. trachomatis* immunoglobulin (Ig) G antibodies in women with a history of miscarriage has been observed (*2*,*3*). *Chlamydophila abortus* is the most common etiology of abortion in ruminants (*4*) and can also cause miscarriage in pregnant women exposed to infected animals.

*Waddlia chondrophila*, another *Chlamydiales*, is a new abortigenic agent in bovines (*5*,*6*). This obligate intracellular bacterium was isolated from aborted fetuses in the United States (*7*) and in Germany (*8*). A serologic study further supported the abortigenic role of *Waddlia* in bovine species (*6*). Moreover, infection of bovine fetuses with *Waddlia* was associated with their deaths within 2 weeks (*6*). In this study, we tested women with sporadic and recurrent miscarriages for antibody reactivity against *Waddlia* and compared seroprevalence with that found in a control group of women with uneventful term pregnancies.

## The Study

From July 2004 to March 2005, we studied 69 women with sporadic miscarriages (SM), 200 women who had suffered from recurrent miscarriages (RM), and 169 control women who had had uneventful pregnancies (Table 1). The RM group comprised women who had >3 miscarriages and who had attended the Recurrent Miscarriage Clinic of St Mary’s Hospital, London, the largest specialist referral center in Europe.

**Table 1:** Characteristics of study patients according to miscarriage history*

Characteristics	Controls (n = 169), no. (%)		Sporadic miscarriages (n = 69), no. (%, p value†)		Recurrent miscarriages (n = 200), no. (%, p value†)
Age, y					
Median (IQR, p value†)	30.4 (25–35)		31.7 (27–36, 0.18)		35.4 (31–39, <0.001)
No. pregnancies					
1	90 (53.3)		32 (46.4)		0
2	46 (27.2)		19 (27.5, 0.67‡)		0 (0, <0.001‡)
>2	33 (19.5)		18 (26.1)		200 (100)
Mean (SD, p value†)	1.8 (1.1)		2.1 (1.5, 0.23)		5.2 (3, <0.001)
Parity					
0	0 (0)		49 (71)		113 (56.5)
1	115 (68.1)		11 (15.9, <0.001‡)		59 (29.5, <0.001‡)
2	31 (18.3)		4 (5.8)		19 (9.5)
>2	23 (13.6)		5 (7.3)		9 (4.5)
Mean (SD, p value†)	1.54 (0.95)		0.52 (1.01, <0.001)		0.64 (0.9, <0.001)
Miscarriages					
Early (<12 weeks)	0		52 (75.4, <0.001)		196 (98, <0.001)
Late (>12 weeks)	0		13 (18.9, <0.001)		51 (25.5, <0.001)
Stillbirth (>24 weeks)	0		4 (5.8, <0.001)		11 (5.5, 0.002)
Alive child	169 (100)		21 (30.4, <0.001)		78 (39, <0.001)
Ethnicity					
White	80 (47.3)		34 (49.3, 0.79)		132 (66, <0.001)
Black	35 (20.7)		10 (14.5, 0.27)		22 (11, 0.01)
Asian	31 (18.3)		18 (26.1, 0.18)		34 (17, 0.74)
Other	22 (13)		6 (8.7, 0.35)		8 (4, 0.002)
Contact with animals	29 (17.2)		11 (15.9, 0.82)		70 (35, <0.001)
Cat	18 (10.7)		5 (7.3, 0.42)		37 (18.5, 0.035)
Dog	15 (8.9)		6 (8.7, 0.97)		28 (14, 0.13)
Fish	1 (0.6)		0 (0, 1)		8 (4, 0.043)
Rodents	0		2 (2.9, 0.08)		7 (3.5, 0.017)
Other	3 (1.8)		0 (0, 0.63)		12 (6, 0.041)
*Chlamydia trachomatis* (IgG titer>50)	15 (8.9)		9 (13, 0.33)		39 (19.5, 0.004)
Positive serology for *Waddlia* spp.					
IgG titer >64	12 (7.1)		22 (31.9, <0.001)		66 (33, <0.001)
IgG titer >128	6 (3.6)		10 (14.5, 0.002)		29 (14.5, <0.001)
Western blot *Waddlia* IgG					
<1 band	12 (7.1)		22 (31.9, <0.001)		63 (31.5, <0.001)
<2 bands	6 (3.6)		15 (21.7, <0.001)		51 (25.5, <0.001)
<3 bands	3 (1.8)		9 (13, <0.001)		28 (14, <0.001)
<2 specific bands§	1 (0.6)		9 (13, <0.001)		22 (11, <0.001)

*Controls, women with uneventful pregnancies; IQR, interquartile range. †Compared with control group. ‡p value for ordered categories (Pearson χ^2^ test). §According to *Waddlia* hyperimmune mouse serum, bands at 61-, 55-, 53-, 45-, 41-, 38-, and 30-kDa are considered specific for *Waddlia* spp.

Immunofluorescence tests were performed (*9*); we used *W. chondrophila* strain ATCC VR-1470 as antigen and we screened sera at a 1:64 dilution with FluolineH (bioMerieux, Marcy l’Etoile, France). Antigen was isolated as described (*10*,*11*). Mice and rabbit anti-*Waddlia* antibodies were used as positive controls with a fluorescein-conjugated anti-mouse and anti-rabbit globulin. Sera that exhibited an Ig titer >64 were tested for IgG and IgM reactivity by using corresponding anti-human Ig fluorescein (FluolineG or FluolineM, bioMérieux) and serial 2-fold dilutions of serum. IgG and IgM positivity cut-offs were >1:64 and >1:32, as proposed for other chlamydia-like organisms. One hundred women had an anti-*Waddlia* IgG titer >64 (Table 1). Seroprevalence was higher for patients who had sporadic (31.9%) and recurrent (33%) miscarriages than that for women who had had uneventful pregnancies (7.1%, p<0.001 when comparing either SM or RM groups to controls). One woman had a positive IgM titer of 64 and an IgG titer of 512.

**Figure 1:**
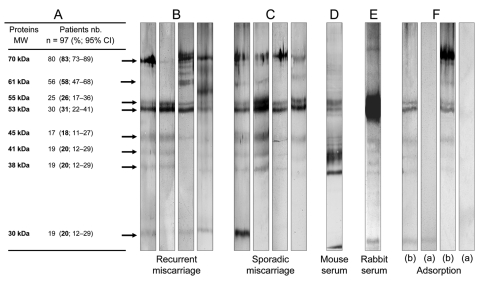
Western blot analyses. A) Molecular weight and frequency of IgG reactivity of *Waddlia* proteins, as determined by Western blots. B) and C) Four representative Western blot patterns of *Waddlia* IgG positive sera from recurrent and sporadic miscarriage groups. D) and E) Representative Western blot pattern of positive control (*Waddlia* hyper-immune mouse and rabbit serum, respectively). F) Western blot performed with *Waddlia* IgG positive sera, taken from patients who had miscarried before (b) and after (a) adsorption with *Waddlia* antigen. MW, molecular weight; nb, number.

To confirm the specificity of immunofluorescence, we performed Western blot analyses on all *Waddlia*-positive sera samples. Western blot was performed (*9*), but *Waddlia* was used as the antigen. A polyclonal peroxidase-labeled anti-human IgG (Dako, Glostrup, Denmark) was used as a secondary antibody. The presence of anti-*Waddlia* IgG antibodies was confirmed by Western blot in 97 of the 100 positive samples by immunofluorescence (Table 1; Figure 1, panels B and C). By using *Waddlia* spp. hyperimmune mouse and rabbit sera and corresponding peroxidase-conjugated anti-mouse/rabbit sera, we obtained similar patterns of 61-, 55-, 53-, 45-, 41-, 38- and 30-kDa proteins (Figure 1, panels D and E). Antibody reactivity against the 55-, 53-, 45-, 41- and 38-kDa proteins disappeared after adsorption with 10^8^ *Waddlia* antigen for 48 hours, which demonstrated the specificity of the antibody response (Figure 1, panel F).

For further statistical analyses, only patients whose samples were positive for *Waddlia* spp. by immunofluorescence and confirmed by Western blot were considered seropositive for *Waddlia* spp. (n = 97). In all age groups, the rate of *Waddlia* seropositivity was higher in patients who had miscarried than in those who had not (data not shown). Moreover, most women with anti-*Waddlia* antibodies did not exhibit serologic reactivity against *C. trachomatis* (Table 2).

**Table 2:** Characteristics of patients in the study according to their *Waddlia* serostatus

	*Waddlia* negative* (n = 341)	*Waddlia* positive† (n = 97)	p value
Age, y			
Median (interquartile range)	33 (28–37)	36 (31–39)	<0.001
No. pregnancies			
1	107 (31.4)	15 (15.5)	
2	62 (18.2)	16 (16.5)	0.006‡
>2	172 (50.4)	66 (68)	
Mean (SD)	3.2 (2.6)	4 (3.1)	0.005
Parity			
0	108 (31.7)	54 (55.7)	
1	156 (45.8)	29 (29.9)	0.001‡
2	49 (14.4)	5 (5.2)	
>2	28 (8.2)	9 (9.3)	
Mean (SD)	1.04 (1.04)	0.7 (1)	<0.001
Miscarriages			
Early (<12 wk)	170 (49.9)	81 (83.5)	<0.001
Late (>12 wk)	47 (13.8)	17 (17.5)	0.36
Stillbirth (>24 wk)	12 (3.5)	3 (3.1)	1
Alive child	228 (66.9)	40 (41.2)	<0.001
Ethnicity			
White	184 (54)	62 (63.9)	0.08
Black	52 (15.3)	15 (15.5)	0.96
Asian	70 (20.5)	13 (13.4)	0.11
Other	29 (8.5)	7 (7.2)	0.68
Contact with animals	76 (22.3)	34 (35.1)	0.011
Cat	42 (12.3)	18 (18.6)	0.12
Dog	35 (10.3)	14 (14.4)	0.25
Fish	6 (1.8)	3 (3.1)	0.42
Rodent	7 (2.1)	2 (2.1)	1
Other	12 (3.5)	3 (3.1)	1
Noninfectious miscarriage causes	97 (28.5)	19 (19.6)	0.08
Autoimmune disease	19 (5.6)	1 (1)	0.09
Hypertensive disorder	8 (2.4)	3 (3.1)	0.71
Endocrine pathology	13 (3.8)	2 (2.1)	0.54
Anatomic abnormalities	4 (1.2)	2 (2.1)	0.62
Additional serologies (titers)			
*Chlamydia trachomatis* (IgG>50)	49 (14.7)	14 (14.3)	0.98
*Clamydophila pneumoniae* (IgG>64)	161 (47.2)	38 (39.2)	0.16
*Cp. psittaci* (IgG>1/64)	20 (5.9)	6 (6.2)	1

*Patients with a *Waddlia* immunoglobulin G (IgG) titer <64 (n = 338) or not confirmed by Western blot analysis (n = 3). †Patients with a *Waddlia* IgG titer >64 and confirmed by Western blot analysis. ‡p value for ordered categories (Pearson χ^2^ test).

**Figure 2:**
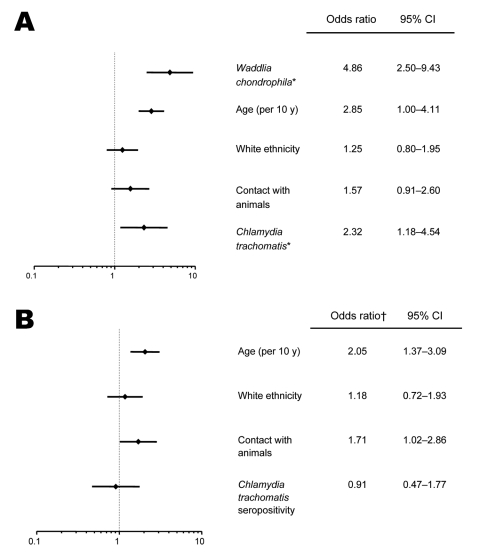
Multivariate analyses. A) Multivariate analysis adjusted for all variables listed in this figure and showing the independent association of age, positive *Waddlia* serologic results and positive *Chlamydia trachomatis* serologic results for women who had had a miscarriage. B) Multivariate analysis adjusted for all variables listed in this figure and showing the independent association of animal contact and advancing age with serologic evidence of *Waddlia* infection. *, seropositivity; †, odds ratio for *Waddlia* seropositivity.

In a multivariate logistic regression adjusted for age, ethnicity, contact with animals and *C. trachomatis* serostatus (Figure 2), miscarriage (SM/RM) remained strongly associated with *Waddlia* seropositivity (odds ratio [OR] 4.9, 95% confidence interval [CI] 2.5–9.4). In this model, miscarriage was also independently associated with age (OR 2.9, 95% CI 2.0–4.1) and *C. trachomatis* seropositivity (OR 2.3, 95% CI 1.2–4.5). Additional multivariate models confirmed the association between *Waddlia* IgG seropositivity and miscarriage, with ORs ranging from 4.9 to 6.2.

Noninfectious causes of miscarriage have also been recorded (Table 2). When studying only the 322 patients without known concurrent conditions, the strong correlation between *Waddlia* seropositivity and miscarriage was still present (68/195 [34.8%] of patients who had had a miscarriage vs. 10/127 [7.9%] of patients who had not miscarried had anti-*Waddlia* antibodies; p<0.001).

*Waddlia* seropositivity was associated with early miscarriage (p<0.001, Table 2). No difference in ethnicity was observed between those who were IgG positive for *Waddlia* spp. and those who were negative. Moreover, there was no difference in *C. trachomatis*, *Chlamydophila pneumoniae*, or *Cp. psittaci* seropositivity between women who had anti-*Waddlia* antibodies and those who did not, which suggests that *Waddlia* antibodies do not cross-react with *Chlamydiaceae*. Moreover, only 7 (1.6%) of 438 patients had a *Parachlamydia* IgG titer >64. With such a low *Parachlamydia* prevalence, cross-reactivity with this chlamydia-like organism is unlikely to explain the high *Waddlia* seroprevalence observed in the miscarriage groups.

Women who were IgG seropositive for *Waddlia* spp. were more likely to have had contact with animals. In a multivariate logistic regression model adjusted for age, ethnicity, and *C. trachomatis* serostatus, those who had had previous contact with animals were more likely to exhibit anti-*Waddlia* antibodies (OR 1.7, 95% CI 1.0–2.9, Figure 2). In this model, *Waddlia* IgG–positive serologic test results were also independently associated with age (OR 2.1, 95% CI 1.4–3.1).

## Conclusion

This study demonstrates a strong association between the presence of *W. chondrophila*–specific IgG antibodies and early fetal loss. Cross-reactivity with other microorganisms seems an unlikely explanation for our results because *W. chondrophila* did not react with monoclonal or polyclonal antisera directed against *Rickettsia*, *Coxiella*, *Wolbachia*, *Anaplasma*, and *Chlamydia* spp (*8*,*12*). We did not detect any cross-reactivity of *W. chondrophila* with *C. trachomatis*, *Cp. pneumoniae*, and *Cp. psittaci*. Moreover, the molecular weights of Waddlial immunoreactive proteins obtained by Western blot are clearly different than those reported for *C. trachomatis* or *Cp. pneumoniae* (*13*).

With the exception of 1 patient who had IgM, only IgG antibody reactivity against *W. chondrophila* was observed. Because IgG antibodies may persist for years after an acute infection has resolved (*3*), the underlying mechanism of miscarriage due to *W. chondrophila* may involve reactivation of a latent asymptomatic waddlial infection, endometrial damage from a past waddlial infection, or an immune response to an epitope shared by a waddlial and fetal antigen, as proposed for *C. trachomatis* (*3*).

The association we found between contact with animals and positive serologic results for *Waddlia* spp. raises the zoonotic potential of this bacterium. This hypothesis is further supported by the range of hosts for *Waddliaceae*. Other modes of transmission are possible (e.g., contaminated water) because free-living amebae may serve as hosts for *Waddlia* spp (*14*). and are widespread in water networks (*15*). *Waddlia* spp. may also be transmitted through ingestion of contaminated cow milk. Finally, *Waddlia* spp. might represent a sexually transmitted disease.

Further investigations are urgently needed to define how *Waddlia* spp. infection may be acquired. To confirm the role of *W. chondrophila* in miscarriage, it will be important to isolate this intracellular bacterium from miscarriage products or confirm its presence in the placenta by immunohistochemistry or PCR. This may be difficult to achieve, if, as suggested for *C. trachomatis* (*3*), *W. chondrophila* causes miscarriage indirectly, e.g., through increased cytokine production or molecular mimicry with fetal antigens. To our knowledge, this work provides the first evidence that *W. chondrophila* may be implicated in human fetal death.
